# Detracking Autoencoding Conditional Generative Adversarial Network: Improved Generative Adversarial Network Method for Tabular Missing Value Imputation

**DOI:** 10.3390/e26050402

**Published:** 2024-05-04

**Authors:** Jingrui Liu, Zixin Duan, Xinkai Hu, Jingxuan Zhong, Yunfei Yin

**Affiliations:** 1 College of Computer Science, Chongqing University, Chongqing 400044, China; 2 Chongqing University-University of Cincinnati Joint Co-op Institute, Chongqing University, Chongqing 400044, China; 3 School of Pharmaceutical Sciences, Chongqing University, Chongqing 401331, China; 4 College of Mechanical and Vehicle Engineering, Chongqing University, Chongqing 400044, China

**Keywords:** generative adversarial network, conditional label, detracking autoencoding, tabular data, imputation

## Abstract

Due to various reasons, such as limitations in data collection and interruptions in network transmission, gathered data often contain missing values. Existing state-of-the-art generative adversarial imputation methods face three main issues: limited applicability, neglect of latent categorical information that could reflect relationships among samples, and an inability to balance local and global information. We propose a novel generative adversarial model named DTAE-CGAN that incorporates detracking autoencoding and conditional labels to address these issues. This enhances the network’s ability to learn inter-sample correlations and makes full use of all data information in incomplete datasets, rather than learning random noise. We conducted experiments on six real datasets of varying sizes, comparing our method with four classic imputation baselines. The results demonstrate that our proposed model consistently exhibited superior imputation accuracy.

## 1. Introduction

In the era of big data, the occurrence of missing values may involve multiple factors, including human error, technical issues, and limitations in the data collection process. For instance, in the process of data collection using industrial instruments, due to environmental factors like severe vibrations or electromagnetic interference, measuring devices may not function correctly, struggle to collect accurate data, or gather data with errors [1]. Moreover, long input data exceeding storage capacity can also lead to the loss of local variables. For industrial time-series data, partial information loss can lead to the disappearance of implicit periodicity or trend-based historical changes, making subsequent accurate analysis and prediction difficult. For example, in equipment operation monitoring, missing data at certain time points could prevent timely detection of faults or prediction of maintenance needs. In the case of cross-sectional data, partial information loss can lead to the loss of feature data, thereby increasing the difficulty of data analysis and interpretation, impacting business decision-making. For instance, in human resource management, missing certain attributes of employees could affect salary structures and performance evaluations. For image and video data, information loss can lead to quality issues and abnormal events. Therefore, imputation algorithms, as a major method of data preprocessing, are essential. They are also widely applied in many other fields, such as genomic sequencing, population surveys, and counterfactual estimation [2,3,4].

The missing mechanism characterizes the relationship between missing and complete data [5]. Assume that $X,P\in R^{m\times n}$ represent the original matrix and missing probability matrix, respectively. Let $p^{\lambda }(j)$ denote the average missing rate of the j-th column in the original data. $W=(w_{1},w_{2},\ldots ,w_{n}),K=(k_{1},k_{2},\ldots ,k_{n}),\lambda =(\lambda_{1},\lambda_{2},\ldots ,\lambda_{n})$ are random numbers in (0,1). Then, various missing mechanisms can be mathematically represented as follows: (1) Missing Completely at Random (MCAR), where data missingness is purely random and not influenced by any variables: $p_{i,j}=p^{\lambda }(j)$; (2) Missing at Random (MAR), where the missingness of data is related to observed values but not to unobserved ones: $p_{i,j}=\frac{p^{\lambda }(j)\cdot m\cdot \exp\left(-\sum_{l<j}w_{l}\lambda_{l}(i)+k_{l}\left(1-\lambda_{l}(i)\right)\right)}{\sum_{s=1}^{m}\exp\left(-\sum_{l<j}w_{l}\lambda_{l}(s)+k_{l}\left(1-\lambda_{l}(s)\right)\right)}$; and (3) Missing Not at Random (MNAR), where the absence of data is related to unobserved values, possibly due to unobserved variables influencing the reason for missingness: $p_{i,j}=\frac{p^{\lambda }(j)\cdot m\cdot \exp\left(-w_{j}x_{i,j}\right)}{\sum_{j=1}^{m}\exp\left(-w_{j}x_{i,j}\right)}\;$. Our method assumes a missing mechanism based on MCAR and focuses on its extension to MAR and MNAR, while discussing the comparative accuracy of our imputation method against others. Beyond the missing mechanism, datasets also vary in terms of missingness rate, sample size, feature dimensions, and data type (numeric, categorical, and mixed). We anticipate that a missing value imputation algorithm will have universality across different scenarios and exhibit superiority over other models.

In addressing missing values within datasets, the mainstream approaches fall into three main categories: no treatment, direct deletion, and imputation methods. For datasets with a small proportion of missing values, direct mining and analysis of the original data containing null values can be performed, utilizing methods such as Bayesian networks [6,7,8]. The direct deletion method involves removing data objects, attributes, or paired variables that contain missing values [9]. In terms of imputation, methods are categorized into statistical techniques and neural network algorithms, with top-tier generative adversarial network (GAN) interpolation belonging to the latter. However, their applicability is limited, primarily to training images [10,11]. Furthermore, for tabular data, a GAN’s generator freely produces data rather than generating specific outputs based on specific conditions. However, most real-world scenarios involve probabilistic one-to-many mappings from inputs to outputs. Additionally, GAN discriminators assess the data as a whole, resulting in their generators’ random inputs having weak correspondence with the data. This can lead to generated data deviating from the main topic, indicating a dominance of global information over local detail.

This paper introduces an enhanced generative adversarial network, termed the detracking autoencoding conditional generative adversarial network (DTAE-CGAN). It incorporates an additional conditional variable, y, allowing the generative adversarial network to extend into a conditional model, guiding the data generation process to ensure that the generated samples correspond with their labels. Furthermore, a detracking autoencoder is introduced, ensuring that the generator G learns not merely meaningless noise data but guarantees point-to-point supervised input and output. We redefine the input structure of hidden neurons, diminishing the self-tracking nature of output neurons to their corresponding input neurons. This assists the network in learning the interrelations among attributes within the dataset. The intermediate latent variables, generated by encoding input data, strengthen the correspondence between the latent variables and the original data, leading to more regulated generated data. By applying three convolutional reductions, the imputed data are then fed into an attention layer to select samples with high relevance, ultimately producing a discrimination matrix of the same dimension as the original data. This approach enables the GAN to learn global and local information in a balanced manner.

Overall, this study makes the following contributions:

- An improved GAN-based imputation neural network is specifically designed for filling missing values in tabular data of various types, demonstrating its generalizability.
- Generative adversarial networks are extended to conditional models, enhancing imputation quality through implicit categorical information in incomplete data.
- The proposed generator–discriminator framework balances the local and global information of missing data.

## 2. Related Work

### 2.1. Statistical Methods

Statistical methods commonly employ means [12], medians [13], modes [14], K-nearest neighbors (KNNs) [15], and singular value decomposition (SVD) [16] for imputing missing values. These approaches are straightforward and widely applicable, particularly to numerical data, yet they often overlook valuable information within the dataset, such as the distribution of missingness and conditional variables. Although methods like Multiple Imputation by Chained Equations (MICE) [3,17], random forests [18,19], and cold deck [20] and hot deck imputation [21] offer practical alternatives, their effectiveness is primarily limited to numerical data [22]. This limitation can lead to the generation of data that lack the quality and richness that could be further enhanced by considering the full spectrum of information available in the dataset, including the underlying patterns of missingness and the relationships between variables.

### 2.2. Neural Network Methods

Existing state-of-the-art neural network imputation methods include recurrent neural networks (RNNs) [23,24] and generative adversarial networks (GANs) [25,26] for filling in missing values. Unlike statistical approaches, artificial neural network imputation relies on the transmission of information through connection weights among a large number of neurons. Each neuron receives input from the previous layer’s neurons, processes it through weighted summation and an activation function, and then outputs it to the next layer. By adjusting the connection weights, artificial neural networks can be gradually optimized to accomplish the task of predicting missing values [27,28]. Compared to statistical methods, this offers a highly effective and practical solution for complex missing data problems (Table 1).

In datasets with sequential relationships, such as time series, RNN imputation can predict missing values in future samples by learning patterns and regularities within the data. While RNNs are utilized for processing end-to-end sequence data, they fall short of capturing the multidirectional dependencies inherent in tabular data and offer little advantage in handling purely numerical datasets. This limitation underscores the need for methods that can adequately address the complex interdependencies and the diverse nature of data found in tabular formats.

GAN-based imputation methods learn the distribution patterns to generate realistic data samples. GAIN [26] utilizes the GAN architecture for imputing missing data frames. However, it confuses categorical and continuous variables during the training process [29]. Variants of GAIN, such as PC-GAIN [30] and GAMIN [31], along with WGAN [32,33], which introduces the Wasserstein distance as loss function, exhibit similar limitations to the original GAIN approach. They typically take the entire data frame as input, focusing on a singular global pattern, which results in overlooking crucial local attributes and information within the data.

## 3. Model Architecture

### 3.1. Problem Statement

In this study, we first define the original data matrix as

$$X={\left(x_{1},x_{2},x_{3},\ldots ,x_{m}\right)}^{T}\in R^{m\times n}$$

Among these, $x_{1}$ to $x_{m}$ are n-dimensional row vectors, representing the number of m samples under n attributes, and R represents the set of real numbers. The matrix containing random missing elements on the original matrix is $\tilde{X}$.

Then, we extract the mask matrix M of the missing data, simulate the distribution of missing and non-missing data, and record the raw and generated data during the training process. The dimension of the mask matrix is the same as that of the original dataset, and the missing identification matrix $M\;\in R^{m\times n}$ corresponding to the missing data is defined as

$$M_{i}=\left\{\begin{array}{l} 1\;\;\;{\tilde{X}}_{i}\;\mathrm{is}\;\mathrm{not}\;\mathrm{NaN}\\ 0\;\;\;{\tilde{X}}_{i}\;\mathrm{is}\;\mathrm{NaN} \end{array}\right.$$

In addition, for the convenience of subsequent training, we convert the missing data type of NAN to random noise z and define a numerical matrix $X^{\prime }$ for input model training.

$$X_{i}^{\prime }=\left\{\begin{array}{l} {\tilde{X}}_{i}\;\;\;{\tilde{X}}_{i}\;\mathrm{is}\;\mathrm{not}\;\mathrm{NaN}\\ z\;\;\;\;\;{\tilde{X}}_{i}\;\mathrm{is}\;\mathrm{NaN} \end{array}\right.$$

### 3.2. Model Structure

Generative adversarial networks typically consist of a generator, G, and a discriminator, D, where G learns and reconstructs the distribution of data, while D assesses the probability that input data originate from real data as opposed to being generated by G.

**Figure 1:**
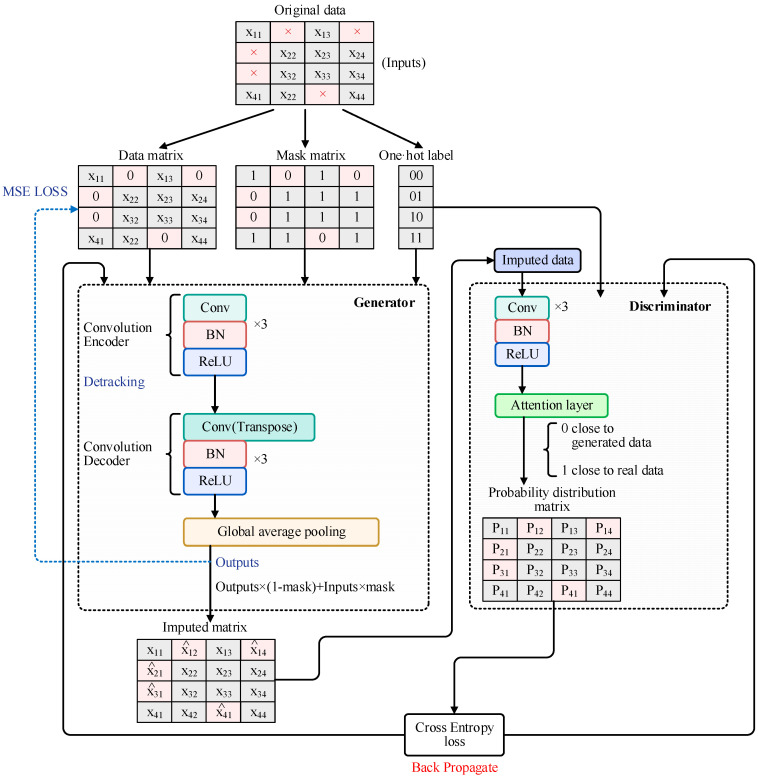
The overall architecture of the model.

However, this model is overly naive and fails to capture the heterogeneity between different subsets or class labels within real datasets [34]. In order to accomplish the imputation task, as shown in Figure 1, the DTAE-CGAN process begins with the combination of the complete data X and the mask matrix M, resulting in data with missing values, denoted as $\tilde{X}=X⊙M$, where the function ⊙ represents element-wise multiplication. Here, M is a binary vector of the same shape as X, with $M_{i}=1$ indicating that ${\tilde{X}}_{i}$ has observed data (not missing) and $M_{i}=0$ indicating that ${\tilde{X}}_{i}$ is missing. Subsequently, $\tilde{X}$ and the label y, as additional information, along with zero-mean Gaussian noise z, are fed into G, resulting in the output data $X^{\prime }=G\left(\tilde{X},(1-M)⊙z|y\right)$. However, we are only concerned with the imputed values when $M_{i}=0$. Further, the imputed matrix $\hat{X}$ obtained from the original data $\tilde{X}$ is $\hat{X}=M⊙\tilde{X}+(1-M)⊙X^{\prime }$.

In DTAE-CGAN, the generator is internally implemented via an autoencoder, which consists of an encoder and a decoder, each equipped with three sets of convolutional layers, batch normalization layers, and ReLU activation functions. The encoder compresses the input missing data into multiple low-dimensional representations, extracting the intrinsic representation of the data; meanwhile, the decoder maps the input missing data to a higher dimension, reconstructing the original input data. Furthermore, this study introduces dynamic filling of missing values through a detracking autoencoder within the generator, aiming to prevent the generator from learning meaningless identity mapping and enhance diversity. In the improved detracking encoder, the computation rule for the k-th node of the first hidden layer connected to the input layer is as follows:

$$O_{kj}^{l}=\sigma (\sum_{l=1,l\neq j}^{s}w_{lk}x_{il}+a_{k})$$

The activation function of neuron is denoted by σ. The weight parameter connecting the l-th node of the input layer to the k-th node of the first hidden layer is represented by $w_{lk}$, and $a_{k}$ is the bias for the k-th node of the hidden layer. In the subsequent h-th hidden layer, the input $x_{ij}$ is also ignored. The calculation rule for the k-th node is

$$O_{kj}^{h}=\sigma (\sum_{l=1}^{m^{h-1}}w_{lk}^{h}O_{lj}^{h-1}+a_{k}^{h})$$

In the subsequent h-th hidden layer, the number of nodes in the previous hidden layer is represented by $m^{h-1}$. The weight parameter $w_{lk}^{h}$ connects the l-th node of the previous hidden layer to the k-th node of the current hidden layer. The output $O_{lj}^{h-1}$ of the l-th node in the previous hidden layer, under this computation rule, is considered. The bias for the k-th node of the h-th hidden layer is denoted by $a_{k}^{h}$. Consequently, the final output $y_{ij}$ disregards the corresponding network input $x_{ij}$ and is instead calculated based on other network inputs. This approach allows for the computation of output values without directly relying on the specific input value at the same position, effectively inferring missing or unavailable information from the available data in the network.

**Figure 2:**
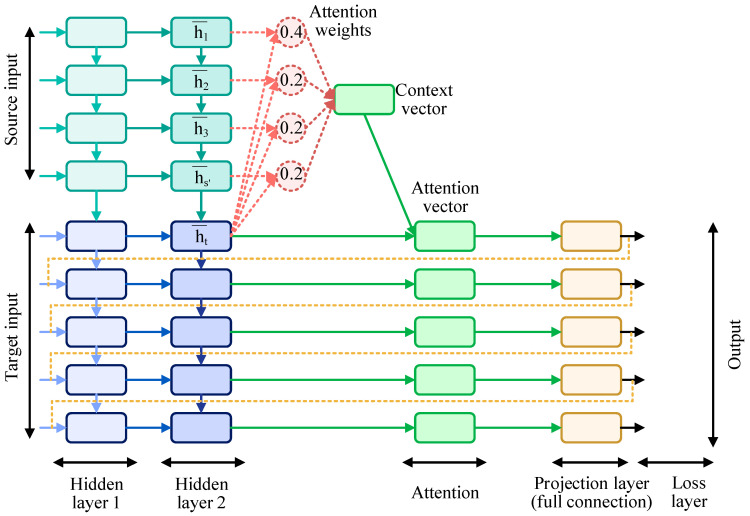
The architecture of deep attention.

The discriminator primarily consists of convolutional layers and attention layers, into which the imputed matrix $\hat{X}$ and label y are fed. After $\hat{X}$ is reduced in dimensionality through the convolutional layers, the attention layer is mainly used to select data with high relevance to the output, grasping local information. This process yields a probability distribution matrix of the same dimension as the original data. With its structure depicted in Figure 2, the computation method is as follows:

The current training state ${\bar{h}}_{s}$ is compared with the discriminator’s encoding target $h_{t}$ to calculate the similarity. This results in an attention probability distribution, represented as attention weights.

$$\alpha_{ts}=\frac{\exp(score(h_{t},{\bar{h}}_{s}))}{\sum_{s^{\prime }=1}^{S}\exp(score(h_{t},{\bar{h}}_{s^{\prime }})}$$

$$score(h_{t},{\bar{h}}_{s})=v^{T}\tanh(w_{1}h_{t}+w_{2}{\bar{h}}_{s})$$

$$c_{t}=\sum_{s}\alpha_{ts}{\bar{h}}_{s}$$

$$\alpha_{t}=f(c_{t},h_{t})=\tanh(W_{c}\left[c_{t};h_{t}\right])$$

v represents the weight vector, and $w_{1}$ and $w_{2}$ are the weight matrices. The context vector $c_{t}$ is the weight sum of attention probability distribution.

Upon completing the calculation of the attention vector $\alpha_{t}$, the data are brought into a fully connected layer, where they are combined with the correctly constructed loss function for classification purposes. In this setup, an output of 1 indicates that the discriminator judges the data produced by the generator to be close to the real data, while an output of 0 is considered to be fake data generated by the generator. This process is repeated. The discriminator D takes as input both the output $\hat{X}$ from the generator G and the conditional label y and outputs a probability matrix that represents the likelihood of $\hat{X}$ being classified as real data $\hat{M}=D\left(\hat{X}|y\right)\in \left[0,1\right]$.

### 3.3. Loss Functions

The loss function of G comprises three parts: generative loss, reconstruction loss, and correlation loss. When optimizing G, the objective is to maximize the probability of $\hat{X}$ being judged as real data by D, which involves minimizing the cross-entropy (CE) loss to deceive the discriminator. The goal is to make the discriminator output values as close to 1 as possible at the corresponding positions where $m_{ij}=0$ in the mask matrix.

$$L_{\mathrm{gen}\;}=-\mathrm{CE}(1,\hat{M})=\sum_{i,j}\left(\left(1-m_{ij}\right)\times \log\left({\hat{m}}_{ij}\right)\right)$$

During the reconstruction process, we minimize the $L_{2}$ distance (i.e., MSE for continuous data) or cross-entropy loss (for binary data) between the non-missing parts of $\hat{X}$ and the corresponding parts in the original data X.

$$L_{\mathrm{rec}}=\left\{\begin{array}{ll} \mathrm{MSE}(X⊙(1-M),\hat{X}⊙(1-M))=\sum_{i,j}{\left(m_{ij}\times x_{ij}-{\hat{x}}_{ij}\times m_{ij}\right)}^{2}, & \mathrm{if}\;x_{ij}\;\mathrm{is}\;\mathrm{continuous},\\ CE(X⊙(1-M),\hat{X}⊙(1-M))=-\sum_{i,j}m_{ij}x_{ij}\log{\hat{x}}_{ij}, & \mathrm{if}\;x_{ij}\;\mathrm{is}\;\mathrm{binary}. \end{array}\right.$$

To maintain the correlation between the local features of the generated data and the original data, we also introduce a correlation coefficient matrix and use the mean squared error to quantitatively measure the difference between the two matrices. In the formula, $\bar{X}$ represents the mean of the current column, and the matrix element $r_{ij}$ denotes the Pearson correlation coefficient between the ith and jth columns of the data.

$$r(X)=\frac{{\left(X-\bar{X}\right)}^{T}⊙(X-\bar{X})}{\|X-\bar{X}\|⊙\|X-\bar{X}{\|}^{T}}$$

$$L_{\mathrm{cor}\;}=E\left[\|r(X)-r(\hat{X}){\|}_{2}^{2}\right]$$

The loss function of D is defined as follows, where λ is a hyperparameter that controls the degree of regularization. An $L_{2}$ regularization term involving the weights $w_{D_{i}}$ of the discriminator is introduced to prevent overfitting.

$$L_{D}=\sum_{j=1}^{N}\left[m_{j}\log({\hat{m}}_{j})+(1-m_{j})\log(1-{\hat{m}}_{j}\right)]+\lambda \|w_{D_{i}}{\|}_{2}^{2}$$

### 3.4. Optimization Goals

Overall, the optimization goal of DTAE-CGAN is

$$\begin{array}{l} \underset{G}{\min}\;\underset{D}{\max}\;V(D,G)=E_{X,M,y,z}\left[\log\left(1-D\left(G\left(X⊙M,z|y\right)\right)\right)+\log D\left(G\left(X|y\right)\right)\right]+\lambda \times \|w_{D_{i}}{\|}_{2}^{2}\\ +L_{\mathrm{gen}\;}+\alpha \times L_{\mathrm{rec}}+\beta \times L_{\mathrm{cor}} \end{array}$$

The decision-making process is as follows: the training objective for the generator is to maximize the probability of the generated samples being identified as “real”, while minimizing the generative error $L_{\mathrm{gen}}$, reconstruction error $L_{\mathrm{cor}}$, and correlation error $L_{\mathrm{cor}}$. This is achieved by the generator function $G(\tilde{X},y,z)$, which takes the missing matrix, conditional label, and noise as input to generate the estimated matrix $\hat{X}$. The training objective for the discriminator is to accurately identify real samples as “real” and generated samples as “fake” [1]. This is accomplished through the discriminator function $D\left(\hat{X},y\right)$, which accepts generator outputs and labels, ultimately producing a distribution matrix $\hat{M}$ representing the probability that the input sample is real data. Through minimizing the loss of the generator and maximizing the loss of the discriminator, the generator and discriminator engage in a competitive interaction. As training progresses, the samples generated by the generator become sufficiently realistic that the discriminator is unable to accurately distinguish between real and generated data. Simultaneously, the discriminator cannot provide additional information to further improve the generator. This signifies the attainment of Nash equilibrium, where both parties are unable to unilaterally modify their strategies to gain better returns. The game theoretic process drives our model to learn effective representations of the data distribution, as shown in Algorithm 1.
**Algorithm 1**. DTAE-CGAN for Missing Value Imputation**Input:**
• **Dataset**

• **Number of epochs**
**Output:**
• **Trained DTAE-CGAN model**

• **Best generator model**

• **Filled missing value in the test set**

• **RMSE value**
1: Load dataset2: Perform standardization3: Split dataset into train and test sets4: Generate random missing data mask5: Defne generator model6: Defne discriminator model7: Defne DTAE-CGAN model using generator and discriminator8: Set best value to positive infnity9: Set test best value to positive infnity10: Set no improvement count to 011: **for** each epoch in range(epochs) **do**12:  Randomly sample generated data from train set13:  Generate noise using normal distribution14:  Train DTAE-CGAN with Adam optimizer15:  Calculate current value and accuracy16:  Calculate filled data and compute test value17:  **if** current value is less than best value and test value is less than best test    value **then**18:      Update best test value19:      Save current generator model 20:  **end if**21: **end for**22: Load best model23: Fill missing data in test set using generator 24: Compute RMSE value

## 4. Experiments and Analysis

The experiment employed the Windows 10 operating system, utilizing the TensorFlow framework with Python 3.10 to conduct neural network training on simulated UCI datasets. NVIDIA CUDA 11.2 was utilized for GPU acceleration. Hardware components for the experiment include dan RTX 3060 Ti GPU, an i5-13400F 4.3 GHz CPU, and 16 GB of memory, as shown in Table 2.

In the same developing environment, we set the training and testing dataset ratio to 4:1 and applied 5 cross-validations, with each experiment conducted 10 times. Under this training strategy, G and D were alternately trained 1000 times with a learning rate of 0.001. Data were randomly dropped MCAR at different proportions, with missing rates set at 10%, 20%, 30%, 40%, 50%, 60%, and 70%, generating corresponding numerical matrices and mask matrices based on the drop locations. Additionally, unless specifically stated, data uniformly had 20% missing rates. We report RMSE and post-classification accuracy as performance metrics, along with their mean and standard deviation across the 10 experiments on the test set.

To ensure a fair comparison with GAIN and its variants, we set the hyperparameters of the loss function as α=200 and β=1.5 and applied λ=0.3 for the three larger datasets, namely Wine, Magic, and Letter. To increase the penalty term and prevent overfitting, we adjusted λ to 0.6 for other smaller-scale datasets. This setup was empirically found to provide good performance in adversarial training [29,30].

### 4.1. Data and Preprocessing

To validate the effectiveness of our method, we conducted experiments on six actual UCI datasets (Seeds, Breast, Wine quality, Magic telescope, Letter, and Skin). As shown in Table 3, seeds and Breast represent relatively small-scale datasets, whereas Wine, Magic, Letter, and Skin denote large-scale datasets. These datasets are numerical, categorical, and do not contain missing values. Missing data were artificially generated in a random manner.

For categorical variables, we treated them as labels of different samples to facilitate classification tasks. For all numerical variables in each column, we applied min–max normalization to linearly transform the data into the $\left[0,\;1\right]$ interval, making them suitable for further training in the model.

### 4.2. Evaluation Metrics

In this experiment, the root mean square error (RMSE) was utilized to measure the difference between the predicted value and the actual value. For the predicted matrix $\hat{X}$ and the data matrix X, we only calculated the error between the imputed values for the missing parts and their true values.

$$RMSE(X,\hat{X})=\sqrt{\frac{1}{\sum_{i,j}\left(1-m_{ij}\right)}\sum_{i,j}\left((1-m_{ij})\times x_{ij}-(1-m_{ij}\right)\times {\hat{x}}_{ij}{)}^{2}}$$

In addition to intuitively measuring the difference between actual and predicted values, we also applied the completed data after imputation to classification tasks. To fairly assess the model’s accuracy in predicting classes in classification problems, and to reflect the potential negative impact of filled missing data on data classification, we used the same random forest classifier for all tests. In binary classification problems, TPs (true positives) represent the number of positive samples correctly predicted as positive, TNs (true negatives) represent the number of negative samples correctly predicted as negative, FPs (false positives) represent the number of negative samples incorrectly predicted as positive, and FNs (false negatives) represent the number of positive samples incorrectly predicted as negative. The accuracy of the binary classification model, denoted as rfc.score (random forest classification score), ranges from 0 to 1, where 1 indicates a perfect prediction and 0 indicates a completely incorrect prediction. The formula is as follows:

$$rfc.score=\frac{\mathrm{TP}+\mathrm{TN}}{TP+TN+FP+FN}$$

For multi-class classification problems, an extended version of rfc.score was used. Considering the true positives, true negatives, false positives, and false negatives for each category, let C be the number of categories. The elements of the confusion matrix M, where $M_{ij}$ represents the number of samples that belong to the actual category i and are predicted as category j, can be used to extend the calculation method of the binary rfc.score to multi-class situations.

$$rfc.score=\frac{\sum_{i=1}^{C}M_{ii}}{\sum_{i=1}^{C}\left(\sum_{j=1}^{C}M_{ij}\right)}$$

### 4.3. Experimental Results

#### 4.3.1. Comparison on Algorithms with Different Missing Probabilities

To quantitatively measure the imputation performance of our model under various missing rates, we conducted comparative experiments by benchmarking it against classic statistical algorithms (mean [12], SVD [16]) and variants of GAIN (PC-GAIN [30], WSGAIN-CP [35]) applied to different datasets.

**Figure 3:**
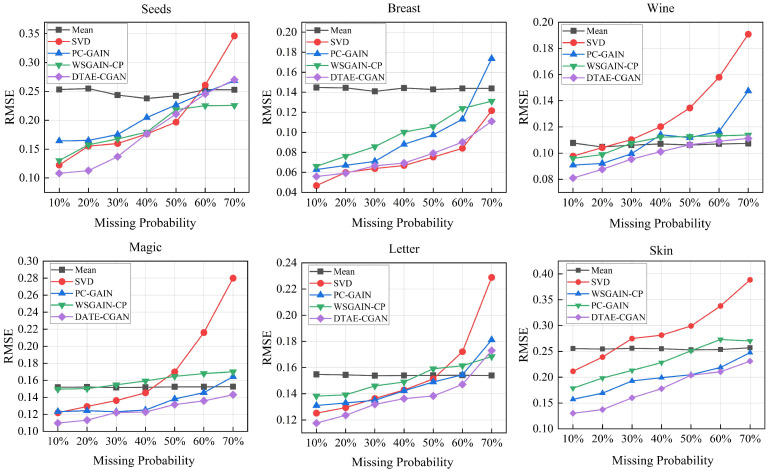
Results of RMSE analysis for each dataset.

Apart from mean imputation, which maintained stability across all missing rates, the RMSE of other methods tended to increase with the rising missing rates, as shown in Figure 3. SVD and WSGAIN-CP performed better on datasets with smaller data volumes, such as Seeds and Breast. As the data volume increased, the advantages of DTAE-CGAN began to emerge. In the Wine, Magic, and Letter datasets, our method outperformed others across MCAR missing rates from 10% to 50%, indicating our model’s superiority in performing imputation tasks at low to medium missing rates. However, at very high missing rates, our model is at risk of degenerating to a general GAN, making it difficult to discern performance differences among other methods under these conditions. The outcome in the Skin dataset reinforces the scalability of our model for large-scale data scenarios, where the sample scale exceeds 200,000.

#### 4.3.2. Ablation Experiment

Table 4 explores the impact of different modules on the imputation metrics by adding or removing corresponding components of our model. With precision, we systematically eliminated the attention module, the detracking autoencoder, and the conditional label from our detracking autoencoding conditional generative adversarial network in Figure 1 one by one so as to ascertain the effectiveness of each module. This includes directly measuring the discrepancies between real and imputed samples using the RMSE metric and post-imputation accuracy, with the latter evaluating the performance of imputed data under the same classification model (random forest classifier).

The performance of DTAE-CGAN was enhanced with the simultaneous introduction of the detracking autoencoder, attention module, and label information. More precisely, by incorporating additional conditional variables, the performance of the baseline model improved by 0.9–8.8% across six datasets, as measured by RMSE. The individual introduction of the attention mechanism and detracking autoencoder did not significantly enhance model performance. However, their synergistic action, when introduced together compared to CGAN alone, resulted in a performance increase of 9.6–23.2%. The performance of post-imputation classification, calculated as the rfc.score, also showed similar results (Table 4).

#### 4.3.3. Influence of Samples and Feature Dimensions

In the experiments above, our model demonstrated strong adaptability to datasets with different characteristics and varying levels of missing data. To gain a better understanding of the model, we conducted experiments with different sample sizes and feature dimensions using the Letter and Breast datasets, respectively.

**Figure 4:**
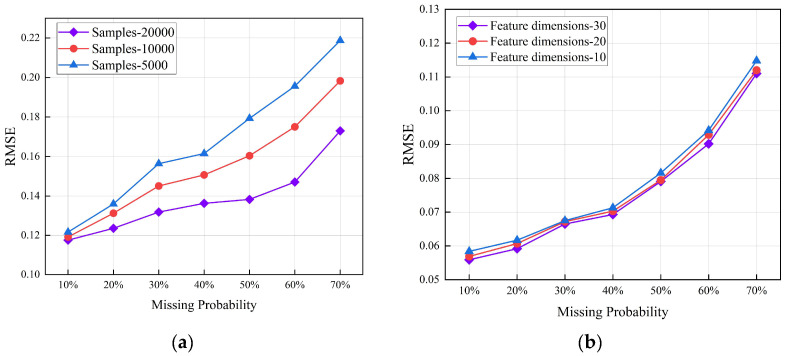
The data filling effect for (**a**) number of samples; (**b**) number of feature dimensions.

Figure 4a demonstrates that as the number of samples increased, the RMSE continuously decreased, indicating better performance. This is due to the large number of parameters of DTAE-CGAN that require optimization, hence necessitating a substantial number of training samples. 

Figure 4b reveals the robustness of our model to changes in feature dimensions when the missing level remained constant. Despite utilizing a deep learning architecture, it still exhibited excellent performance in handling low-dimensional data.

#### 4.3.4. Computational Cost Efficiency

**Figure 5:**
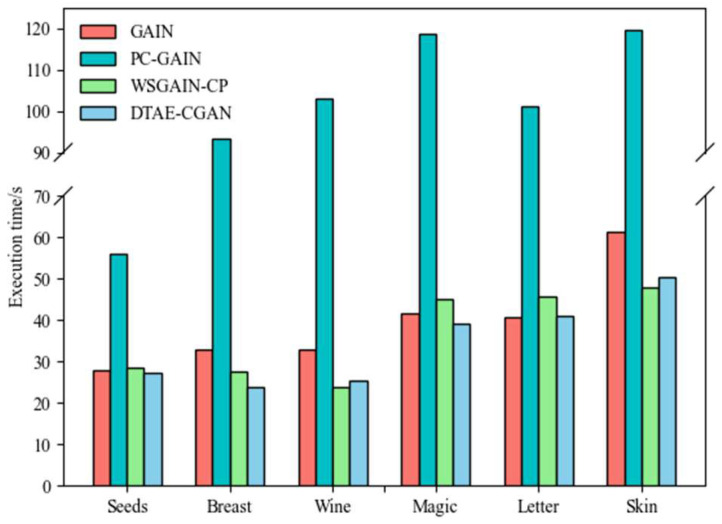
Execution time of different GAIN variants.

A fair comparison of the imputation time of our model with GAIN and other variants was conducted. Each model underwent 5-fold cross-validation, and the results were averaged over 10 experiments. As illustrated in Figure 5, our model exhibited the highest efficiency across the majority of datasets, with improvements ranging from 1.52% to 16.09% over the second-ranked model. In the Letter dataset, the performance of DTAE-CGAN was comparable to that of GAIN. With a few exceptions, DTAE-CGAN demonstrated a slightly slower performance than WSGAIN-CP on the Wine and Skin datasets. Among all datasets, PC-GAIN demonstrated the lowest efficiency.

#### 4.3.5. Extension to MAR and MNAR Mechanism

The different missing mechanisms have a crucial impact on the imputation performance of the model. By setting intervals and constraints for the presence of missing values, we can simulate which features or samples have missing values or remain intact. For the MAR mechanism, based on the arrangement of the data, we refer to [19] and specify (1) the one-third pattern, where missing values can only appear in the middle one-third of the feature columns and (2) the two-thirds pattern, where missing values can only appear in the first one-third and last one-third of the feature columns (Table 5 and Table 6). For the MNAR mechanism, following [22], we randomly removed data points in numeric attributes that were less than the median of that attribute (Table 7).

In the 12 tests conducted under the two different scenarios of MAR, the DTAE-CGAN model ranked first in 9 instances, consistently outperforming others on datasets like Wine, Magic, Letter, and Skin, which are of a larger scale, indicating a superior imputation performance. Under the MNAR missing mechanism, SVD exhibited an absolute advantage over the GAIN model, suggesting that generative adversarial models do not necessarily exhibit top-notch imputation performance under all missing distributions. Depending on the characteristics of the dataset, appropriate imputation methods should be chosen. Nevertheless, compared to variants of GAIN, our model still demonstrates significant superiority.

## 5. Discussion

Imputation schemes based on generative models have achieved top-tier standards in previous research [1,26,36]. However, GANs are limited in their application and have weaker capabilities in capturing the implicit information within data. By optimizing the model framework, we propose that DTAE-CGAN can utilize missing variables within categorical datasets. At low to medium missing rates, our model effectively leverages a detracking autoencoder and attention mechanisms to enhance the learning of feature variables, overcoming the adverse effects of training noise, thereby improving the accuracy of imputation. Moreover, the model exhibits good robustness to features, and the presence of a large number of data samples with strong correlations is also beneficial for driving its learning. This is because there are numerous parameters to be optimized in each module of DTAE-CGAN, requiring a massive amount of data for training to achieve better generalization and learning improvement.

As for the computational cost, the outstanding performance of DTAE-CGAN can be attributed to its unique approach, which avoids generating a hint matrix through a hint generator. But similarly to WSGAIN-CP, DTAE-CGAN utilizes only two-layer neural networks. Additionally, we feed the original data matrix into the generator and guide the actual generation process through the use of autoencoder-based reduction, rather than relying solely on noise variables z as in GAIN. The clustering and pre-training phases of another variant PC-GAIN significantly extend the overall running time. In contrast, this approach significantly enhances imputation efficiency.

For MCAR, where missing data are unrelated to other variables, and for MAR, where missing data are related to features of known data, repeated training of the generator and discriminator can yield better estimates of missing data, as their absence is random. However, in the case of MNAR, DTAE-CGAN may face greater challenges, as generated data must reflect unobserved features, potentially requiring more complex model structures or additional prior knowledge to handle MNAR.

It is worth noting that as the missing rate increases, the number of training epochs required for convergence also increases. There is a risk that our model may degrade to a conventional GAN. When the missing rate is 0.5, the generated data closely resembles the original distribution. However, when the missing rate exceeds 0.6, it becomes challenging to reduce the error. Nonetheless, our model still maintains a slight advantage over other models in such scenarios. This may be attributed to the fact that filling the missing parts directly with random noise z disrupts the learning of correlations. When the missing rate is very high, most of the input to G consists of random noise, and as a result, G and D no longer significantly outperform classical imputation methods. Regardless of whether the missing rate is high or low, the generated data consistently approximate the original distribution. The limitation arises when there is an excessive number of missing data, preventing the effective use of additional information to reconstruct the non-missing portions.

Hence, the advantages of our model are as follows:

- It provides a model framework dedicated to imputing missing values in tabular data, which is suitable for various data types.
- The imputation performance of this model is superior compared to other baselines, and it demonstrates stronger robustness to missing rates and feature dimensions.

The potential improvements for our model are as follows:

- The model structure requires further refinement to accommodate various missing mechanisms, particularly MNAR.
- It needs to be further refined to impute missing values at high missing rates.

## 6. Conclusions

We propose a novel tabular data imputation method, DTAE-CGAN, which is capable of learning the implicit information of sample labels while considering both global and local information. Experiments on six real UCI datasets demonstrated that our method can flexibly handle different data types, missing rates, sample sizes, and feature dimensions. It performs well in various settings, providing a reference method for the field of missing value imputation.

Future work will further investigate the impact of different missing mechanisms. Additionally, we plan to enhance the model’s accuracy in datasets with high missing rates. Moreover, incorporating domain-specific prior knowledge into the imputation process represents a significant direction for improvement.

## Figures and Tables

**Table 1:** Comparison of mainstream imputation methods.

Imputation Method	Category	Scope of Application	Advantages	Limitations
No treatment	~	Any missing mechanism, any data type	Simple and fast	Low accuracy
Direct deletion	~	MCAR	Simple and fast	Easy to lose useful information
Mean	Statistical method	MCAR, numerical	Simple	Introducing deviation
Hot deck imputation	Statistical method	MAR, any data type	Simple	Influenced by the definition of similarity criteria and the sample selection process
Cold deck imputation	Statistical method	MAR, any data type	Simple, full consideration of the periodicity of data	The filling effect depends on the quality of historical data
Multiple imputation	Statistical method	Any missing mechanism, any data type	Focusing on the uncertainty of missing values under different mechanisms	Does not have the same theoretical definition as other methods, difficult to capture real data patterns
KNN	Statistical method	MCAR and MAR, numerical or categorical	Suitable for small datasets and those with low missing rates	Inaccurate high-dimensional data or high missing rates, dependent on the selection of K value
SVD	Statistical method	MCAR and MAR, numerical	Extracting the main features while retaining the most important information	Sensitive to number of samples and dimensions
Random forest	Statistical method	MCAR and MAR, any data type	Accurate and stable	Poor interpretability
RNN	Deep learning	Any missing mechanism, any data type (time series)	Considering the time dependence between variables, especially in time series data	Unable to effectively utilize distant historical data
GAN	Deep learning	Any missing mechanism, any data type	Small dependence on samples in existing datasets, highest accuracy	Large memory usage and long training time, sensitive to random noise

**Table 2:** Experimental environment.

Hardware Environment	Software Environment
CPU: i5-13400F 4.3 GHz	Programming language: Python
Memory card: 16 GB	Programming platform: TensorFlow 2.6.0
Graphics card: NVIDIA CUDA 11.2	Operating system: Windows10

**Table 3:** Dataset properties.

Datasets	Categories	Samples	Features
Seeds	3	210	7
Breast	2	569	30
Wine	2	6497	11
Magic	2	19,020	10
Letter	24	20,000	16
Skin	2	245,057	3

**Table 4:** Imputation performance conducted by control variable (mean ± Std).

Datasets	Variants	RMSE	rfc.score
Seeds	DTAE-CGAN	0.1127 ± 0.0083	0.9761 ± 0.0135
	DTAE-CGAN w/o attention	0.1353 ± 0.0068	0.8809 ± 0.0117
	DTAE-CGAN w/o DTAE	0.1189 ± 0.0091	0.9523 ± 0.0133
	DTAE-CGAN w/o DTAE&attention	0.1265 ± 0.0105	0.9662 ± 0.0149
	DTAE-CGAN w/o DTAE&attention&labels	0.1404 ± 0.0116	0.9285 ± 0.0152
Breast	DTAE-CGAN	0.05949 ± 0.0045	0.9951 ± 0.0022
	DTAE-CGAN w/o attention	0.07498 ± 0.0035	0.9942 ± 0.0010
	DTAE-CGAN w/o DTAE	0.07680 ± 0.0058	0.9939 ± 0.0028
	DTAE-CGAN w/o DTAE&attention	0.07746 ± 0.0060	0.9912 ± 0.0022
	DTAE-CGAN w/o DTAE&attention&labels	0.07960 ± 0.0067	0.9824 ± 0.0056
Wine	DTAE-CGAN	0.0876 ± 0.0007	0.9796 ± 0.0052
	DTAE-CGAN w/o attention	0.0959 ± 0.0011	0.9661 ± 0.0064
	DTAE-CGAN w/o DTAE	0.0965 ± 0.0013	0.9523 ± 0.0049
	DTAE-CGAN w/o DTAE&attention	0.1016 ± 0.0014	0.9500 ± 0.0045
	DTAE-CGAN w/o DTAE&attention&labels	0.1027 ± 0.0019	0.9546 ± 0.0062
Magic	DTAE-CGAN	0.1132 ± 0.0029	0.7799 ± 0.0035
	DTAE-CGAN w/o attention	0.1347 ± 0.0014	0.7721 ± 0.0056
	DTAE-CGAN w/o DTAE	0.1427 ± 0.0045	0.7436 ± 0.0047
	DTAE-CGAN w/o DTAE&attention	0.1386 ± 0.0038	0.7526 ± 0.0033
	DTAE-CGAN w/o DTAE&attention&labels	0.1496 ± 0.0054	0.7515 ± 0.0031
Letter	DTAE-CGAN	0.1236 ± 0.0003	0.5715 ± 0.0032
	DTAE-CGAN w/o attention	0.1372 ± 0.0003	0.5393 ± 0.0040
	DTAE-CGAN w/o DTAE	0.1420 ± 0.0014	0.5484 ± 0.0028
	DTAE-CGAN w/o DTAE&attention	0.1367 ± 0.0018	0.5502 ± 0.0030
	DTAE-CGAN w/o DTAE&attention&labels	0.1397 ± 0.0027	0.5055 ± 0.0064
Skin	DTAE-CGAN	0.1372 ± 0.0002	0.9642 ± 0.0017
	DTAE-CGAN w/o attention	0.1552 ± 0.0005	0.9579 ± 0.0026
	DTAE-CGAN w/o DTAE	0.1587 ± 0.0011	0.9610 ± 0.0019
	DTAE-CGAN w/o DTAE&attention	0.1806 ± 0.0008	0.9517 ± 0.0030
	DTAE-CGAN w/o DTAE&attention&labels	0.1915 ± 0.0015	0.9488 ± 0.0039

**Table 5:** Imputation performance of MAR mechanism (one-third, mean ± Std).

Datasets/Methods	Mean	SVD	PC-GAIN	WSGAIN-CP	DTAE-CGAN
Seeds	0.2491 ± 0.0034	0.1686 ± 0.0052	0.1092 ± 0.0093	0.1258 ± 0.0023	0.1236 ± 0.0098
Breast	0.1013 ± 0.0060	0.0675 ± 0.0015	0.0873 ± 0.0095	0.0791 ± 0.0037	0.0707 ± 0.0049
Wine	0.0829 ± 0.0088	0.0768 ± 0.0028	0.0926 ± 0.0066	0.0602 ± 0.0026	0.0524 ± 0.0080
Magic	0.1429 ± 0.0081	0.1334 ± 0.0042	0.0978 ± 0.0071	0.0894 ± 0.0060	0.0764 ± 0.0041
Letter	0.1610 ± 0.0059	0.1665 ± 0.0034	0.1403 ± 0.0052	0.1481 ± 0.0039	0.1320 ± 0.0028
Skin	0.1658 ± 0.0072	0.0902 ± 0.0050	0.0515 ± 0.0066	0.0639 ± 0.0044	0.0475 ± 0.0045

**Table 6:** Imputation performance of MAR mechanism (two-thirds, mean ± Std).

Datasets/Methods	Mean	SVD	PC-GAIN	WSGAIN-CP	DTAE-CGAN
Seeds	0.2477 ± 0.0073	0.1446 ± 0.0084	0.1368 ± 0.0132	0.1261 ± 0.0077	0.1335 ± 0.0146
Breast	0.1558 ± 0.0069	0.0613 ± 0.0031	0.0762 ± 0.0087	0.0846 ± 0.0043	0.0829 ± 0.0020
Wine	0.1179 ± 0.0085	0.1220 ± 0.0055	0.1145 ± 0.0083	0.1053 ± 0.0054	0.1005 ± 0.0034
Magic	0.1518 ± 0.0050	0.1531 ± 0.0082	0.1456 ± 0.0069	0.1567 ± 0.0070	0.1328 ± 0.0027
Letter	0.1516 ± 0.0033	0.1180 ± 0.0077	0.1409 ± 0.0042	0.1207 ± 0.0039	0.1099 ± 0.0033
Skin	0.2005 ± 0.0048	0.2070 ± 0.0029	0.1554 ± 0.0051	0.1693 ± 0.0025	0.1461 ± 0.0029

**Table 7:** Imputation performance of MNAR mechanism (mean ± Std).

Datasets/Methods	Mean	SVD	PC-GAIN	WSGAIN-CP	DTAE-CGAN
Seeds	0.2785 ± 0.0045	0.0955 ± 0.0091	0.1130 ± 0.0040	0.1362 ± 0.0132	0.0766 ± 0.0079
Breast	0.1432 ± 0.0028	0.0384 ± 0.0087	0.1418 ± 0.0057	0.0623 ± 0.0242	0.0924 ± 0.0090
Wine	0.1163 ± 0.0036	0.0682 ± 0.0108	0.1003 ± 0.0074	0.1252 ± 0.0194	0.0924 ± 0.0052
Magic	0.1609 ± 0.0056	0.0895 ± 0.0150	0.1125 ± 0.0022	0.1944 ± 0.0106	0.1238 ± 0.0074
Letter	0.1952 ± 0.0083	0.1201 ± 0.0123	0.1447 ± 0.0065	0.1390 ± 0.0117	0.1338 ± 0.0094
Skin	0.3142 ± 0.0042	0.1537 ± 0.0071	0.1686 ± 0.0028	0.1396 ± 0.0125	0.1243 ± 0.0157

## Data Availability

The data presented in this study are available on request from the corresponding author.
